# Metaphilosophy of Mind: how Do Minds Investigate Minds? Refutation of the Theocentric View

**DOI:** 10.1007/s12124-016-9362-6

**Published:** 2016-09-24

**Authors:** Konrad Werner

**Affiliations:** Institute of Philosophy, Cognitive Science Department, Jagiellonian University in Kraków, Grodzka 52, 31-044 Kraków, Poland

**Keywords:** Mind, Philosophy of mind, Metaphilosophy, Philosophy of cognitive science, Knowledge

## Abstract

I shall propose metaphilosophy of mind as the philosophy of mind *investigating mind*. That is to say, I pose the question of how knowledge of mind provided by cognitive science, broadly construed, is constrained by the epistemic position of the knower, i.e. by the very fact that it is *undertaken by a mind*. Here I would like to propose a *minimal* framework, based on two distinctions: (i) the standard one between empirical and conceptual analysis; (ii) a new one, between the internal questions of mind and the boundary questions of mind. I shall then combine these distinctions to arrive at several ways of investigating the mind, the brain and cognition. On this ground, I will discuss the notion of epistemological theocentrism as outlined by Henry Allison and argue against the perspective I call theocentric philosophy of mind. From this angle I will be able to address skepticism which cannot be defeated but actually can be, as I put it, *disarmed*. Finally, metaphilosophy of mind based on the abovementioned distinctions elicits a perspective that is not sufficiently delineated by cognitive scientists and philosophers: *empirical* way of addressing the *boundary* questions of mind.

## Introduction

How is *knowledge of mind* possible? Metaphilosophy of mind is introduced here as an attempt to answer this question and by the same token to increase clarity with respect to the problems and results of cognitive science and related fields. As such, metaphilosophy of mind covers a broad area of investigation. In this essay a *minimal* framework is proposed, based on two distinctions: (i) the standard one between natural and conceptual analysis; (ii) a new one, drawing on the idea of boundary questions. However, “new” means rather a *new formulation* than a genuinely *new thought*. I shall then combine these distinctions to arrive at several ways of investigating the mind and cognition. On this ground, I will discuss the notion of epistemological theocentrism as outlined by Henry Allison (2006) and argue against the perspective called theocentric philosophy of mind.

Here is the plan: First it is explained briefly why a suitable meta-reflection on mind should be undertaken (Metaphilosophy of Mind and the Epistemic Position section). Secondly, the distinction between natural and conceptual analyses as applied to the investigations of mind is brought up (in Empirical and Conceptual Analyses of Mind section). These two parts can be regarded as introductory. The main body of this essay starts from the Introduction of Internal Questions and the Reason why we Need More than that section in which the notions of empirical domain, conceptual framework, and internal questions within a given framework are outlined. Having this done, I will carry on to argue that we need more than internal questions. Namely, we have to come up also with boundary questions which are not supposed to address this or that particular issue within the conceptual framework, but rather they define and delimit the framework itself. All these considerations draw on the ideas already widely known, though they are rarely combined in the way pursued here. Next (Boundary Questions and Architecture Questions section), this essay elaborates more on the conception of a boundary question, and finally arrives at the point where a relatively new idea is brought forth. Namely, two groups of boundary questions are distinguished: *proper boundary questions* and *architecture questions*, putting stress especially upon the latter. Having this scaffold set up, I bring forth (Two More Perspectives on Mind section) the distinction between *internal questions of mind* and *boundary questions of mind*. Now, the natural-conceptual split and the internal-boundary split establish in conjunction the minimal conceptual framework( Combinations: The Conceptual Scaffold of Minimal Metaphilosophy of Mind section) which discerns several metaphilosophical positions, including skepticism, methodological exclusivism, and, most importantly, theocentrism. According to metaphilosophical theocentrism, which rarely becomes explicit, we are actually able to gain the perfect, thus, in a sense, divine access to reality, enabling us to acquaint it *from nowhere*, disregarding our perspectives and cognitive limitations. The last part (Theocentric Philosophy of Mind & Metaphilosophy of Mind Disarms The Skeptic and Elucidate The Proponent of Theocentrism sections) is dedicated to the criticism of theocentric metaphilosophy of mind. Reductive materialism turns out to be a form of theocentrism, meanwhile, as I hold, the prospects of a non-theocentric naturalism has not been sufficiently scrutinized by cognitive scientists and philosophers.

## Metaphilosophy of Mind and the Epistemic Position

Metaphilosophy is usually conceived of as philosophy *of* philosophy (see e.g. Rescher 2006; Williamson 2007). Thus metaphilosophy of mind should be described as *philosophy of* the philosophy of mind. However, this essay addresses not only purely *philosophical* investigations of mind, therefore in order to make my attitude and objectives more precise, let me put it as follows: metaphilosophy of mind is the philosophy of mind *investigating mind*. That is to say, I pose the question of how knowledge of mind is constrained by the epistemic position of the knower, i.e. by the very fact that it is *undertaken by a mind*. In particular, how human knowledge *of* cognition is constrained by the *capacities of human cognition*, viewed in the broader context of evolution, social relations, language, etc. Human epistemic position is thus constrained by the sum of all factors – from the capacity of the sense organs to culture – that determine the *way the world appears to* humans.

The idea of epistemic position (see Werner 2016) shall become clearer over the course of these considerations. Meanwhile, in order to give the reader some basic sense of it, let me cite the cognitive biologist Ladislav Kovac, who writes:

The limits of the human mind, its possibilities and constraints, imposed by contingencies of evolution of the species, seem to be insurmountable. Due to them, our reality, a model of the world, is species-specific. (Implying that other species construct their own species-specific reality.) They confine us to the world of medium dimensions and low complexity (Kovac 2000: 54)

Our model of *the way in which we create models* is also species-specific. In other words, Kovac concludes, “our world of consciousness is a phenomenon of the brain, but our brain is also a phenomenon of the brain” (Kovac 2000: 55).

## Empirical and Conceptual Analyses of Mind

Metaphilosophy of mind, as presented here, starts from the standard distinction: The contrast between scientific investigation, i.e. empirical analysis, often relying on a mathematical apparatus, and conceptual analysis, undertaken in the humanities, including philosophy. This distinction was most likely already established by Plato or Plato’s Socrates, who in the *Phaedo* claims that we should shift our attention to *words*, which possibly means *meanings* or *concepts* (Plato 2009). This is because – as he likely believed – concepts usually mirror the order of the world and at the same time somehow reveal the origin of this order.^1^

Today we obviously do not believe such a Socratic shift to be plausible if conceived of as the *substitution* of one kind of inquiry – the empirical – with another, purely conceptual. But the distinction as such stays valid. The opposite substitution – when empirical analysis replaces the conceptual (take for instance Willard Van Orman Quine’s program: Quine 1969) – was once quite popular; still, philosophers tend to reaffirm their own conceptual task (see Jackson 2000; Poli 2010; Harper 2012; Fine 2012).

Hence, the following two perspectives should be distinguished with respect to investigations of mind:

*Empirical* analysis of mind and its material basis – the brain, the entire nervous system, and the entire body together with its environment. This is the usual approach for neuroscience, as well as experimental psychology, among others. It may also be called a *natural* analysis.

*Conceptual* analysis of mind. Here one asks not so much about the mechanisms underpinning mind, but seeks to understand *what* a mind is, and *what* constitutes its *relationship* to the reality (on the difference between “what” questions and “how” questions see Fine 2012).

Roughly, the empirical/natural analysis may be called a causal story and the other one a conceptual story (see Fodor 1975). The status and character of natural analysis is of course a topic of philosophical, methodological and logical debates, nevertheless its significance is generally unquestioned. That is why these issues will not be developed here. The *difference itself* shall not be addressed too – it is assumed as plausible and uncontroversial. However, it should be stressed that these two perspectives or methods must not be regarded as genuinely separated; it is held here only that the clarifying conceptual distinction is valid.^2^

## Introduction of Internal Questions and the Reason why we Need More than that

Let me introduce some basic notions:

*The empirical domain* is the world around me, the world I live in, the world present to me. At the beginning it is sufficient to define it ostensively – if you want to know what the empirical domain is, look around. As Mark Rowlands nicely puts it, “to say that an item is empirical is to say that it is the sort of thing that, in its nature, can be an object of consciousness” (Rowlands 2003: 206). Inside the empirical domain, we distinguish objects, facts, processes, events, etc.

“Concept” is a term of art, understood in diverse ways depending on the discipline. On the one hand concepts are conceived of as “the constituents of thoughts” (Margolis and Laurence 2014) that constitute propositional content (Peacocke 1995). On the other hand there is a tendency to treat them as tools (Rosh 1978), and a tendency to speak of conceptual *representations* rather than concepts themselves (Goldstone and Kersten 2003; Carey 2009). Concepts themselves are not the proper topic of this essay, so it is assumed here that:

*A concept* is an intellectual tool for making distinctions in the empirical domain; e.g. I see the moving objects outside my window as birds. Note that this characteristic does not presuppose anything about the representational, classical or non-classical, definitional or prototypical *nature* of concepts, but only focuses on their *cognitive* function. Maybe it would be even more appropriate to speak not so much of concepts but rather of *possessing concepts*.

Now, concepts form networks or webs of connections. We impose these *conceptual frameworks* on reality, and it is only within such frameworks that we are able to pose questions and observe regularities (one might argue that all regularities are *products* of conceptual frameworks, not something realized in reality itself; see Ajdukiewicz 1934, Fleck 1935, Kuhn 1962; also Putnam 1987, 1988).

Within this context, Rudolf Carnap (1950) distinguishes *internal questions* from *external questions* and by the same token criticizes philosophy:

If someone wishes to speak in his language about a new kind of entities, he has to introduce a system of new ways of speaking, subject to new rules; we shall call this procedure the construction of a linguistic framework for the new entities in question. And now we must distinguish two kinds of questions of existence: first, questions of the existence of certain entities of the new kind within the framework; we call them internal questions; and second, questions concerning the existence or reality of the system of entities as a whole, called external questions. (…) this [external- KW] question is raised neither by the man in the street nor by scientists, but only by philosophers. (...) And it cannot be solved because it is framed in a wrong way. To be real in the scientific sense means to be an element of the system; hence this concept cannot be meaningfully applied to the system itself (Carnap 1950: 21 – 22)

According to Carnap, one is able to plausibly question the existence of any object or fact only within a particular conceptual or linguistic framework, whereas philosophers attempt to do this outside of any such schema. Carnap’s thesis can be generalised, and as such it does not concern only questions of *existence* – in this view, all philosophical questions are external, whereas all questions of objects and facts are internal, and *only* internal questions are questions of facts and objects. If so, then external questions posed by philosophers cannot refer to the empirical domain, and therefore traditional philosophical theses cannot reveal any order *inside* the empirical domain. Thus, they are useless.

Let A, B, C... refer to given conceptual frameworks. Carnap supposes that for any given question, it is posed either in A, or in B, C, etc. (in other words, it belongs to the sum of A, B, C,…), or it is posed *outside of any framework* and is a meaningless utterance. However, for Carnap the boundary between A and B, and most importantly, the boundary between the sum of A, B, C… on the one hand and the uncountable sum of all meaningless expressions on the other, is fixed and unproblematic. In other words, for Carnap the *limit* of the domain of rational thought is transparent – he does not focus his attention on it, as if he could see both domains - the rational and the meaningless - from an external point of view. Hilary Putnam writes:

What Carnap is trying to do in “Empiricism, Semantics and Ontology,” it would seem to both Quine and Wittgenstein, is to find an *external standpoint from which to condemn external questions as meaningless*. To distinguish a sense that “Chairs exist” or “Material objects exist” has *within the language*, an “internal” sense, from a pseudo-sense which can only be characterized from outside the language, is precisely to miss the point that there is no standpoint available to us outside the language (…) (Putnam 2008: 8)

Putnam stresses what I call the epistemic position – if all cognitive, intellectual activities are trapped in conceptual frameworks, then an overview of *these very frameworks* is trapped as well. We cannot assume “that one can stand within one’s language and outside it at the same time” (Putnam 1990: 23). Putnam rejects in this way the “God-Eye View”, that is to say the “epistemic ideal of achieving a view from an ‘Archimedean point’ – a point from which we can survey observers as if they were not *ourselves*, survey them as if we were, so to speak, *outside our own skins*...” (Putnam 1990: 17).

Putnam’s criticism is apparently in step with Henry Allison’s proposal, and his reading of Immanuel Kant, singling out the *theocentric* approach to knowledge prevailing throughout the history of epistemology, according to which “human knowledge is to be measured and evaluated in terms of its conformity (or lack thereof ) to the norm of a putatively perfect divine knowledge” (Allison 2006: 114). This means *perfect access to reality*, disregarding any limitation, context, aspect, mode of presentation, point of view, etc. We might add, following Thomas Nagel (1986), that epistemological theocentrism endorses the prospect of a view *from nowhere*. And this *ideal knowledge* has been set up as the *ideal of* human cognition, actually unattainable but yielding epistemic norms, by philosophers since Parmenides and Plato, through Descartes (1996) and his followers (see also Gasparyan 2015). In Allison’s reading, Kant’s (1999) *Critique of Pure Reason* is the downright refutation of epistemological theocentrism.^3^ Meanwhile. it turns out that even skeptics such as David Hume are committed to theocentrism. This is because “while denying the possibility of the kind of knowledge to which the rationalist typically pretends, they share the underlying assumption that this is what genuine cognition would be like, if it were attainable by beings such as ourselves. In this methodological respect, then, they likewise adhere to the theocentric paradigm” (Allison 2006: 114). Meanwhile, the only possible knowledge – says Allison and Allison’s Kant – is the knowledge *of* the world *from the perspective of the world*, so to speak.

In this context, Carnap’s criticism might be regarded as purporting to abandon theocentrism by putting stress upon the plurality of linguistic frameworks. However, at the same time, Carnap unwittingly endorses theocentrism by proclaiming the existence of the robust limits of these frameworks, as if he could *see* them all from the God-Eye perspective.^4^ Meanwhile, if all we know is gained from the downside of the world, then our grasp of the limits cutting-off our local epistemic position *is also constrained by this very local epistemic position*.

Thus boundaries between conceptual frameworks and their limits are not merely given, but must be actively *marked out* from the *inside*, so to speak.^5^

Therefore it seems that apart from questions *within* conceptual frameworks, there is also the question *of the boundaries* between frameworks and, crucially, of the boundary between all conceptual frameworks on the one hand, and *the meaningless* or, as Ludwig Wittgenstein (1922) would say, *the unspeakable* on the other. Thus we also need questions about the *limits* of cognitive capacities. In other words, there are questions aimed at targets within the boundaries (limits) of a given conceptual framework (internal questions), and questions aimed at the *boundaries (limits) themselves* (However, by saying this I am also no more aligned with Putnam’s view, as he would probably reject the very idea that such limits *could* and *should* be marked out). The latter seem to be external as long as they are simply *not internal*, but on the other hand they cannot be called merely “external” if this means “aiming at something *outside* the boundaries of conceptual schemata”. They are neither about something inside nor about something outside; the distinction between *outside-inside* is their target.

Hence, the idea of the metaphilosophy of mind that is being put forward here ensues from Allison’s and from Putnam’s stress upon our *local epistemic position*. Metaphilosophy of mind is about the limits or boundaries of the conceptual frameworks *capturing* the local mind, *imposed by* the local mind. By highlighting locality I want to underline the fact that these investigations are being undertaken *from the inside*; in other words metaphilosophy of mind does not target *mindedness as such*, the alleged *essence* of being *a* minded creature (an animal, a Martian, an angel or God) and limitations essential to *any possible* minded creature. It targets human and other at least partially accessible animal minds. In other words it strives to account for the limitations imposed *by them* on *their* studies *of themselves* (imposed *by us* on *our* studies *of ourselves*). Recall Kovac’s remark: The brain is the phenomenon *of* the brain.

## Boundary Questions and Architecture Questions

Questions targeting the limits or boundaries of conceptual frameworks shall be called *boundary questions*. This essay can hardly explore all issues pertaining to them, especially how they are embedded in the philosophical tradition (in Aristotle, in the medieval theory of transcendentals, in Kant and in Husserl among many others; see Kołodziejczyk 2006), but the two general targets of boundary questions as they are understood here, shall be distinguished.

First there is the most general issue of what makes the empirical domain intelligible; that is to say, what makes the world around us *eligible to become the material for human intellectual activity*. Some philosophers suppose that there must be frameworks other than the internal-conceptual ones; more fundamental factors enabling intellectual access to the world, resulting in knowledge and deliberate action; factors which, so to speak, *situate the empirical domain as a whole* within the *space of reasons* (the term introduced by Sellars 1956 and elaborated by McDowell 1994). That is to say, within the space of discourses, arguments, norms, contents, values, predictions, etc. In order to highlight this issue I shall speak of *the intelligible empirical domain* from now on, meaning the empirical domain *as introduced into the space of reasons*. The emphasis on intelligibility is needed since there is no reason for *assuming* that the data provided by senses are *necessarily* introduced to the space of reasons; apparently they *are* but it still the task of a philosopher to ask *how* this *introduction* is possible; *why* it is the case; *what* makes the data in question intellectually accessible.

So, the boundary questions are dedicated to problemize the very intelligibility of empirical domain; i.e. how the domain is brought to the space of reasons. As an example, let me mention that according to John McDowell, the first or the initial capturing of the world within the intellectual framework is brought forth with ethical norms. McDowell writes that human being are “initiated” into the space of reasons “by ethical upbringing, which instills the appropriate shape into their lives. The resulting habits of thought and action are second nature” (McDowell 1994, p. 84). Hence, in my understanding, McDowell’s ideas can be recognized as ensuing from the awareness of boundary questions.

Another very interesting conception of this kind has been proposed by Sebastian T. Kołodziejczyk (2006, 2009). In his view, the first “gates” of mind, so to speak, through which all data must pass in order to become material for further *intellectual* processing, are the so-called *basic metaphysical beliefs*, which are spontaneous, mostly unexpressed and *seemingly* trivial beliefs referring to the world around us. These beliefs bring the world into view for any activity undertaken by the human intellect.^6^ Kołodziejczyk writes:

... a set of beliefs which are prior to any beliefs the human mind is able to support. These beliefs I will call “basic metaphysical beliefs”... A provisional list of them may be as follows:

1. there exists something
2. something is recognized as a thing that has features
3. there is more than one thing; things differ from each other
4. things are knowable for the human intellect
5. things are desirable for the human will

The [basic metaphysical beliefs] listed above are provisional in the sense of their number and form. It is possible to imagine a more precise formulation and a longer list, e.g. with modal statements (Kołodziejczyk 2009: 187).

In other words, (1) expresses the *spontaneous act* of grasping *something that exists*; we could hardly direct our attention toward items which are not regarded as existing in some way. Hence, on the most fundamental level *the intelligible empirical domain* is set up as the domain of *beings*. Meanwhile, (2) expresses the act of grasping *content*. We could hardly direct attention toward an item having no characteristics, no qualities at all – toward, so to speak, pulp. Hence, the intelligible empirical domain is set up by (2) as the domain of *qualified* beings; etc. It is clear that Kołodziejczyk seeks to tackle a boundary problem that, again, can hardly be even expressed if we had only internal questions in our intellectual toolbox.

Now, when the empirical domain is already brought into the space of reasons – by ethical engagement as proposed by McDowell or by Kołodziejczyk’s basic metaphysical beliefs, or in some other manner – there is still the question of how the empirical domain brought into the mind’s view is *arranged*. This question then targets the overall *architecture* of the empirical domain as captured within the space of reasons. In other words, it is not so much about what makes the world as a whole intelligible, but rather, once the world *is* intelligible, about how it is *organized* at the most general level. I call these boundary questions the *architecture questions* whereas the former group I call the *proper boundary questions*.

In order to give the reader a sense of what is meant here by architecture questions, let me put it as follows: We are likely to admit that internal questions of science refer to facts, objects, processes, etc. But it should not come as a surprise that there is no distinct *scientific* discipline investigating FACTS, OBJECTS, PROCESSES, etc. as such, that is to say the general notions setting up the furniture of the empirical domain. Scientific questions are obviously more specialized: For example, what is the mechanism of brain plasticity in the barrel cortex. However, this case may provoke a radical skeptic who might ask why we define the world around us as a domain of *facts*, *objects* or *substances* and *processes* interacting with one another *causally*, instead of defining it in some other manner? Why are FACT, SUBSTANCE, PROCESS, EVENT, RELATION, CAUSATION, etc. the slots in our minds into which we put all the incoming data? How do these slots work? Apparently they are not usual concepts like DOG, BOOK, NEURON or NEUROTRANSMITTER. So what are they?

Before I answer this question let me give two examples pertaining exclusively to the philosophy of mind.

Example 1: The crucial problem of cognitive science is whether it is possible to reduce mental phenomena to physical phenomena. But *what* exactly is it that can or cannot be reduced? Now, the latter question can be understood as an internal one, and answered e.g. by something like “Perceptual states can be reduced; they are nothing more than...” or to use the famous example discussed by Saul Kripke (1980): “Pain can be reduced – it is identical to the C-fiber firing.” A materialist could say that *all* mental states and objects can be reduced to the physical states and objects, and he takes “all” as a universal quantifier encompassing pains, thoughts, emotions, perceptions, etc. However, the question of what can or cannot be reduced can also be understood as an architecture question. How? Just recall that according to the well-known argument by Gilbert Ryle (1949), one makes a fundamental mistake when applying concepts such as OBJECT, STATE or PROCESS to mental phenomena (PHENOMENON should also be included on this list). Ryle seems to be saying something like “Wait a minute! What do you mean by OBJECT here?”, that is to say he wants to elicit someone’s account of the *architecture* of the domain in question. People are accustomed to think as follows, he says: If thinking is not a *physical* process, then it must be some other type of *process*. A materialist disagrees but he accepts the general framework. He says: No, thinking in fact *is* a physical *process*. “Wrong!” – says Ryle. Applying *the very notion of a process* one automatically misrepresents the domain of inquiry. Hence – the mental slots PROCESS, STATE, OBJECT, etc., do not fit the mental domain itself. The argumentation of the late Wittgenstein (1953) runs in a similar vein. We can see that *the way in which the mind-body problem is formulated* – before one tries to solve it – is a deeply nontrivial issue.

Example 2: It seems that the *mind-body* distinction *itself* might be regarded as problematic. John Searle (1992) argues that both the dualist *and* the reductive materialist are trapped by inadequate vocabulary. This “Cartesian vocabulary” pushes them into a purely virtual struggle between two entirely different kingdoms: The kingdom of conscious life and thoughts, and the kingdom of physical entities. They demand a univocal declaration: Which side of the conflict are you on? However, there is no struggle, says Searle. What he means by “vocabulary” in this context is the way in which the domain of inquiry – the domain of animals having consciousness, to put it simply – is delineated before any internal questions and answers can be given. Thus he is referring to something like the *architecture* of the part of the empirical domain that is targeted by cognitive science. It turns out, Searle says, that the traditional slots, PHYSICAL, PSYCHICAL or MENTAL, deform the view of cognizing living creatures.

To conclude this part, let me give a metaphor that in my view appropriately captures the idea architecture questions. If we think of the *intelligible* (accessible) empirical domain as a building, proper boundary questions target those features of the building that constitute it as a whole, the features that guarantee its stability.^7^ Meanwhile, architecture questions target neither the building as a whole nor what is inside – which is the job of internal questions – but draw our attention to the walls constituting the internal structure of rooms, corridors, stairs, etc. Note that, interestingly enough, walls cannot be said to be inside the room, but they are inside the building itself and they establish the internal structure of rooms.

Hence, it seems that architecture questions target what the philosophical tradition calls *categories.* 8

One might then conclude that if they are about categories, then the introduction of a new term is unjustified. I would like to justify my position by saying that they target those tools possessed by the intellect that are poised to *play the role* of categories.

Now, note how Roberto Poli, who himself amply contributes to the theory of categories, introduces Nicolai Hartmann’s conception:

Categories deal with what is universal and necessary... Categories articulate in particular the *Sosein* of entities; they specify configurations, structures and contents, not forms of existence... Categories specify the fundamental determinations of being; they are principles of being (Poli 2014, §3).

The justification for introducing a new term is the following. Keeping in mind Allison’s and Putnam’s criticisms I do not refer to *being* as long as “being” is opposed to mere “appearance.” Within the empirical domain, thus in the world I live in, I refer via architecture questions to those factors that are *employed* spontaneously, as is the case in everyday life, or by convention (decision), as often in science, to reveal or to impose the internal architecture of this domain. However, nobody should claim that there is only *one universal way* of revealing or imposing this architecture; moreover, nobody, in my view, should claim that this architecture could not be changed.^9^

Therefore, while Hartmann feeds categories to the world, my view goes back to Kant and the Cartesian-Kantian focus on the intellect/mind. *If* we are inside the empirical domain, then all our attempts to uncover, reveal or impose the fundamental structure underpinning the domain as a whole (by posing proper boundary questions) or its internal architecture (by posing architecture questions) are undertaken *from the inside*. So, Aristotle’s and Hartmann’s idea of distilling categories from being is theocentric: We are not in a position to step outside of the empirical domain and, say, take a walk around.

The idea of architecture questions stems from my attempt to formulate a *non-theocentric* metaphilosophy of mind (and a non-theocentric metaphysics as well, but that is not my aim here – see Werner, 2015b, a). As a matter of fact, the metaphilosophy of mind is thought of here as an *ex definitione* non-theocentric enterprise and this claim should become clear in the next sections.

## Two More Perspectives on Mind

What about the question of mind? Taking into account the distinction between internal questions and boundary questions, how are we targeting the mind itself?

Having said all of the above we should now see that in addition to two methods of investigating mind – the natural and the conceptual – there are two ways of questioning mind, and consequently two general ways of thinking about the *place* of mind with respect to the intelligible empirical domain:

The internal question of mind: Mind is here conceived as a *part* of the empirical domain, or an *element of the world in which we live*. This, again, is the question and the perspective naturally employed in the empirical sciences. Mind is of course a product of natural evolution, causally connected with and acting within the physical environment, etc.

The boundary question of mind: Unlike in the internal question, here mind is not an element but rather *a condition of possibility* of the empirical domain *qua* an intelligible, internally organized space of reasons. This attitude, traditionally regarded as *transcendental*, is characteristic *inter alia* of Edmund Husserl’s phenomenology. Dan Zahavi writes:

The world, and more generally, every type of *transcendence,* is relative insofar as the condition for its appearance lies outside itself, namely, in the subject (Zahavi 2003: 48)

In order to give the reader a sense of what the boundary question of mind means, let me also quote an ample fragment from Ludwig Wittgenstein’s *Tractatus*:

5.632 The subject does not belong to the world but it is a limit of the world.

5.633 ...

You say that this case is altogether like that of the eye and the field of sight. But you do not really see the eye.

And from nothing in the field of sight can it be concluded that it is seen from an eye.

5.641 There is therefore really a sense in which in philosophy we can talk of a non-psychological I.

The I occurs in philosophy through the fact that the “world is my world”.

The philosophical I is not the man, not the human body or the human soul of which psychology treats, but the metaphysical subject, the limit—not a part of the world (Wittgenstein 1922: 74 - 75).

Hence, mind captured in the boundary question of mind is like the eye mentioned by Wittgenstein – it neither can be inside the empirical domain, nor straightforwardly outside it (thus straightforwardly outside the limits of meaningful questions, as stressed by Carnap), albeit we could hardly think of any empirical domain disregarding the mind (eye). The empirical domain is always *someone’s* empirical domain – “the world is my world.” If there is no eye (standing here for the mind) the very notion of a field *of vision* (standing here for the empirical domain) makes no sense at all.^10^

The distinction set forth above is crucial for these considerations. Let me then recapitulate it in the following way. When we ask an internal question of mind, we target the mind as located *in* the intelligible empirical domain. From this angle mind is seen as *accompanied by other beings*, as it were, so that we can e.g. measure how fast is someone’s reaction to the stimuli in such-and-such circumstances or we can e.g. conceptually target what does it *mean* that someone *intends* to do such-and-such things. The former job is undertaken by psychologists, whereas the latter is rather taken on by such philosophers investigating intentionality as e.g. Searle. On the other hand, when we come up with a boundary question of mind we target the mind *not* as located in the intelligible empirical domain, but as a factor that determines the actual shape of this domain. Thus, for example when Husserl targets intentionality, he means, unlike Searle, intentionality not as a relation occurring *inside* the perceived world, but as a factor determining how the perceived world as a whole is introduced into the space of reasons; to put it bluntly - as a factor determining *what the world is for us*.

## Combinations: the Conceptual Scaffold of Minimal Metaphilosophy of Mind

Having distinguished several approaches to the mind we can now consider a number of connections:

The internal-*empirical* question of mind: Mind as part of the empirical domain investigated by neuroscience and cognitive psychology, among others. Moreover there is suitable philosophical or methodological meta-reflection as part of which we can investigate, for instance, the ontological commitments of particular theories.

The internal-*conceptual* question of mind: This is conceptual analysis which *stays within the empirical domain*, viewing mind and its actions as parts of it. It is here that we find most of naturalistic, reductionist and non-reductionist philosophy of mind. This perspective naturally coincides with meta-reflection on empirical theories, so that sometimes they can hardly be distinguished. The boundary is blurred, nevertheless *a priori* there is a subtle difference between independent philosophical investigations on the one hand, and philosophical meta-reflection on scientific investigations on the other. Someone might *a posteriori* argue that they are equivalent, but I am not going to discuss this claim here.

The boundary-*conceptual* question of mind: Conceptual analysis which transcends the empirical domain, examining its conditions of possibility – hence also the conditions of possibility of mind *taken as an empirical item or fact*. Here we find Kant (1999), Husserl (1913), Merleau-Ponty (1962) and, in some respects, Wittgenstein (1922), among many others. Recall Husserl’s idea of the transcendental subject, i.e. the “subject considered qua condition for appearance, phenomenality, manifestation” (Zahavi 2003: 48).

The boundary-*empirical* question of mind: Here we *empirically* pose the question of mind conceived of as the limit of *empirical* domain. Surely, this option is the most problematic, at the same the most intriguing, albeit usually not sufficiently distinguished from the internal-empirical perspective. I elaborate on it in Metaphilosophy of Mind Disarms the Skeptic and Elucidate the Proponent of Theocentrism section.

Now, we can imagine once again a radical skeptic who, having the list of options, casts doubt on the very possibility of examining mind. His argument is based on pointing out that all attempts to scrutinize mind are self-referential. He relies on Allison’s and Putnam’s refutations of the theocentric approach. He says: We can neither gain access to reality in itself, beyond the factors determining its *appearance for us*, nor to *these factors in themselves.* 11

*Skepticism* must be differentiated from the position baptized “new mysterianism” by Owen Flannagan (Flanagan 1991). The name is extremely inadequate. The inscrutability of mind proclaimed by the skeptic does not mean that the mystery is so mysterious that any explanation can hardly be imagined. There may be as many opinions on this point as there are people. But the issue is not about our opinions, imagination and exaggerated feelings. Skepticism, even if it is wrong in some respects, is a serious concern. Thus the alleged inscrutability rests on the conceptual, methodological and, generally speaking, cognitive constraints that stem from the fact that minds are investigated *by minds*; it stems from the epistemic position of the knower.^12^

Now, one can agree with the skeptic in some respects, but nonetheless argue that some of the paradigms listed are more promising; some of them may lead to scientific success on a modest scale. Call it *methodological exclusivism*. Now, there is a subtle but crucial difference between the latter and metaphilosophical theocentrism, which I introduced following Allison in the beginning of this paper, thereby making it a backdrop for this reflection. The reflection has now arrived at the point when theocentrism can be reintroduced and targeted against the background of the proposed distinctions.

*Theocentrism* might be treated as a strengthening of the exclusivist’s position, while at the same time differing from it in an interesting way. The theocentric philosopher or scientist chooses neither internal nor boundary question of mind; neither conceptual nor empirical approach; instead he *refuses to accept the distinctions*, or he is not aware of them (or similar ones – I do not claim that the list I provide is the only possible list; nevertheless the proponent of theocentrism refuses to accept *any* list). Hence he says that there is a way of investigating the mind and the world – e.g. one of those listed above – which is not only scientifically plausible, but moreover is the only *adequate account of reality itself*. However, “the only” does not point to a *choice* that he makes, or a refutation of other options; it is rather a *refutation of any list of options*. As such, this is the position appealing to a God-like observer’s perspective.

## Theocentric Philosophy of Mind

I argue that a great part of philosophical investigations of mind is indeed theocentric or at least unconsciously approaches theocentrism. Let me give two instructive examples.

Arguing against Frank Jackson’s (1986) argument from the blind color scientist Mary, Paul Churchland concludes:

What Blind Mary is missing is one common form of knowledge about light: she lacks perceptual/discriminative knowledge of light. And yet, people who have such knowledge are accessing the very same features of reality that she is obliged to access in other ways. The difference lies in the manner of the knowing, not in the nature of the thing(s) known. It is true that no amount of propositional knowledge of light will ever constitute the visual apprehension of light, but that is entirely to be expected. They are different forms of knowledge; they operate with different representational “palettes” insides Mary’s brain. But they both represent, each in its own distinct way, one and the same entirely physical thing: light (Churchland 1997: 131).

Churchland distinguishes between the *manner of knowing* and the *nature of the thing* known. This is not wrong – indeed, it is quite natural. However this distinction is contextual, for instance as when we differentiate between modes of seeing colors by people suffering from *achromatopsia* (one of several forms of color blindness) and the way things *really look.* The way things *really look* is still – as recognized in another context – a manner of knowing (or seeing). Namely, it is the *standard way* of seeing in the standard environment. Churchland seems to use this distinction non-contextually (see also Churchland 1981). He stresses the fact that we can have several ways of representing the same thing, and yet *the* thing still remains *entirely physical*. This emphasis allows me to conclude that “is physical” is not a predicate from the Kantian-Allisonian *downside of the world*, hence from another, possibly the best (as the methodological exclusivist might claim), but still *a way* of representing. Instead it is a predicate from a God-like observer’s language. Let me single it out as “is physical_G_,” and let “G” stand for the divine character of a predicate. Things *in themselves*, seen *from nowhere*, are physical_G_. In reality, *an sich*, there are no thoughts_G_, expectations_G_, feeling_G_ etc.; there are entirely physical_G_ objects_G_ and processes_G_.

In a similar spirit William Lycan argues:

Human and other subjects can have functionally or computationally different states that nonetheless home on the same objective state of affairs, either external or internal. But there are no intrinsically subjective or perspectival facts that are either the special objects of self-regarding attitudes or facts of “what it is like.” There are only states of subjects that both function in a particularly intimate way within those subjects and have the subjects themselves and their other states as inevitable referents. And that, I think, is all there is to “subjectivity” (Lycan 1990: 126).

There are no *subjective facts*_G_. “Subjectivity” refers to the *functional role*_G_ that an *objective fact*_G_ plays exclusively in the *internal computational processing*_G_ of a *particular organism/subject*_G_. There are different *perspectives*_G_ *on* (objective) facts_G_ but there are no *perspectival* facts_G_. However, it seems that according to Lycan observers are able to refer to objective facts *disregarding perspectives* and their functional roles.

The materialistic^13^ argument running along these lines – I mean the theocentric, eliminativist position, not the methodological exclusivism favoring natural analysis – can be generalized in the following way: Firstly, there are only objective, physical entities in the world. Secondly, there are differing ways of referring to these entities; different perspectives, roles; different representations; different languages, e.g. physical predicates and mental predicates (see Davidson 1970). However, the predicate “is physical” used in the first thesis in not *one of* those mentioned in the second thesis; it has special status. This is a *privileged* way of referring. But how is this privileged way freed from all the perspectival constraints of the other ways? Is the privileged way still *a way*? Maybe the privileged way is not *a* way, i.e. the observer’s means of getting something or getting somewhere, but the direct *presence* of reality *offered* to the subject? It seems that this *presentational* or even “*illuminational*” (recall the medieval debates on the sources of knowledge of God) character of materialism is underestimated. But how is this special status possible in the context of what we know from the empirical sciences about species-specific cognition constrained by evolution?

Of course theocentrism does not have to be materialistic. However, admittedly, the example of materialism is currently much more relevant than the examples of dualism or idealism/phenomenalism, i.e. the belief that only *mental contents*_G_ really exist_G_.

## Metaphilosophy of Mind Disarms the Skeptic and Elucidate the Proponent of Theocentrism

Now, the point is that one cannot *argue* in favor of theocentrism, since *arguing* in favor of one option requires the acceptance of a *list of options*, be it the list of metaphilosophical attitudes that has been outlined here or some other list, thus the acceptance of the fact that there are other options that one might choose. Hence, to accept theocentrism means either to be simply unaware of such a list or to perform a Kierkegaardian leap of faith rather than making a choice. If so, then arguing against theocentrism is in fact impossible. The controversy is then *not about arguments* and theses, but rather about the ways of one’s acquaintance with the world; it is about, as Wittgenstein (1953) might put it, *ways of life*. A proponent of theocentrism must therefore *recognize* other ways and his own way as *a* way, as *one of* the ways; his appearance of reality as *one of* possible appearances. We could then repeat after Wittgenstein that philosophy – here metaphilosophy of mind – *consists of elucidations* (see *Tractatus* 4.112) as long as it paves the way for the recognition of one’s own epistemic position.

Surprisingly enough, rejection of the theocentric idea of knowledge of mind concerns skepticism as well. This is because the skeptic is, so to speak, a disappointed proponent of theocentrism. He discovers that a knowledge of mind satisfying theocentric conditions is impossible, but he does not abandon those conditions. Hence, he still endorses the theocentric ideal of knowledge. Now, if one *accepts* the idea of knowledge that is more appropriate for finite minds constrained by their epistemic positions, then skepticism is still not rejected, but it is actually *disarmed*.

## Non-Theocentric Naturalism

While theocentrism does not have to be materialistic or, broadly speaking, naturalistic, there is also no need for naturalism to be theocentric. Hence, there is one more combination that still has to be targeted; the one briefly mentioned; i.e. the boundary-*empirical* question of mind. Is it possible at all? That is to say, can we *empirically* pose the question of mind conceived of as the condition of possibility of *empirical* domain?

Admittedly, cognitive psychology gives us quite convincing *empirical* evidence that the world as we see it, thus the *empirical* domain, is a *product* of complex processing, not a mere copy of reality. This perspective is intensively explored, even though psychologists and sometimes even philosophers may not be fully aware of the distinction between the boundary and the internal inquires. However, as we might have already seen thanks to Kovac, there are other promising domains of inquiry that seem to combine the empirical approach and boundary questions, i.e. that treat some features of the world we live in as essentially correlated with cognition; especially with the *evolution* of cognition – from the simplest forms (as e.g. chemotaxis) up to human culture and science. It is enough to mention the theory of autopoietic systems (see Maturana and Varela 1980); cognitive biology (see Kovac 2000; Fitch 2008; Tommasi et al. 2009), cognitive ethology (Lorenz 1973; Shettleworth 2010), and evolutionary psychology.

Let me recall in this context the distinction between proper boundary questions and architecture questions. Now, aside from a more controversial question of whether the *proper boundary questions of mind* could be addressed empirically,^14^ when it comes to the *architecture questions of mind* there is no a priori reason not to answer them empirically. That would mean to single out and investigate *empirically* the cognitive mechanisms resulting in a given architecture or type of architecture of the *empirical* domain. As a matter of fact, this is exactly what cognitive psychology does, e.g. asking about how we perceive the shapes of things, how we perceptually group them, etc. Let me here also cite the cognitive biologist Tecumseh Fitch (2008) on the cognitive evolution of intentionality, starting from so-called nano-intentionality up to more and more complex forms. This is a good example of what a boundary-*empirical* question of mind *could* be:

the assemblage of multiple neurons together leads to a wholly new level of composite intentionality (let’s call it micro-intentionality) that is constituted by the relations of the nerve cells to one another. This is the crucial transition at which the causal powers that we assign to minds (rather than to bodies) become discernible. These relations provide the most elemental building block of mind per se, because now a specific event of neuronal firing can be properly said to be “about” something external in an immediate synchronic fashion, local to the organism (as opposed to the diachronically-derived evolutionary “aboutness” of the cell we have had up to this point) (Fitch 2008: 170).

Independently of what Fitch himself would like to admit, the quoted passage presents an empirically justified account of *how the empirical domain known to us has arisen over the course of cognitive evolution*. Namely, it turns out that the differentiation between the cognizing subject on the one hand, and the *simultaneously existing* external object on the other, thus the *internal-external* (*mind-world*) dichotomy which is the most fundamental feature, the backbone of our post-Cartesian worldview, *depends on the particular organization and specialization of cells*. It seems clear to me, although it would require an extra investigation, that Fitch’s idea can hardly be understood properly if we approach it in terms of internal questions. After all, he outlines how the architecture of the world as we know it *as a whole* comes about thanks to the architecture of cells. Thus, the fact is that the boundary-empirical approach is currently being undertaken, even if it poses a conceptual challenge.

## Conclusion

I have distinguished several perspectives of investigating mind, and combined them, thereby arriving at several attitudes or metaphilosophical positions. Here are some of the most important points:

By distinguishing the internal and the boundary questions of mind we obtain a clearer picture of what our multidisciplinary studies of mind aim at, and a clearer view of their limitations. First of all, by recognizing these limitations we defend ourselves against the perils of theocentrism which, as a leap of faith, violates the norms of rational inquiry, including, of course, science. Theocentric philosophy of mind is based on an inadequate idea of knowledge, i.e. the idea of knowledge assessed according to the standards of a God-like knower, while the only effectively applicable standards are the human ones, constrained by human cognitive tools; generally speaking, by the human epistemic position.

Secondly, we are able to disarm skepticism as the skeptic turns out to be a disappointed proponent of theocentrism. This means that although we cannot prove that skeptics are wrong, and indeed a great deal of their arguments stay valid, nonetheless we can debunk the theocentric idea of knowledge laid down in their view. Our answer could then take the shape of the following declaration: Yes, we agree that a God-like knowledge is impossible. And unlike you, we *accept* this limitation. So, there is no way you can threaten us.

Moreover, we can see now, quite simply, that all questions and answers being brought up by philosophers (and scientists) should be appropriately ascribed to perspectives just outlined. For example, when Husserl addressed subjectivity what he had in mind was not a phenomenon among different phenomena, namely something mental as opposed to something physical; he referred to what should be deemed (in his view) the condition of possibility of *any phenomenon*; of what I call *empirical domain*. Hence, subjectivity as accounted for by Husserl is a boundary-conceptual problem. Meanwhile, subjectivity is being addressed by contemporary philosophers of mind mostly as an internal-conceptual problem. Now, we find ourselves in trouble when we want to read Husserl from this internal perspective.

Finally, by conjoining the internal-boundary split with the conceptual-empirical one, we are able to elicit an option that has not been sufficiently addressed and scrutinized yet, namely the boundary-*empirical* perspective. Let me finish up this essay by issuing a call saying, firstly, that this perspective of inquiry should really be recognized as a *specific* field, naturally correlated with, yet crucially different from the internal-*empirical* one; secondly - that this boundary-*empirical* way requires and deserves a closer meta-theoretical (thus philosophical, methodological) examination.

## Footnotes

1 According to Plato, this is the pre-physical realm of ideas. Hence – and this is the source, as someone might say, of the peculiarity of philosophical effort – conceptual analysis is implicitly also about reality (in Plato’s view, about its most fundamental level).

2 I am also not saying that philosophical reflection is *only* conceptual. Most philosophical claims are apparently about the world, not about *thinking about* the world. Philosophers, who can hardly *rely on* empirical data (since the credibility of these is, precisely, a philosophical problem; however the idea of an experimental philosophy gains traction; see Knobe and Nichols 2008), search for some other way to stay “in touch” with reality – see for instance the efforts of Edmund Husserl (1913), i.e. his transcendental reduction. However, I am interested exclusively in the *conceptual part* of the philosopher’s task and not in the specific characteristics of his/her method or experience.

3 This means that a subject is able to gain access to the world, which is essentially, as it were, from *the inside* or rather from the downside of the world – as opposed to the extramundane position of a God-like knower. The distinction between *appearance* and the *thing-qua-in-itself* means that subjects always have the world presenting itself in *some* manner instead of the world without presentation (Allison endorses a meta-epistemological version of the two-aspect interpretation of Kant; see also Langton 1998). However, crucially, this does not make it a worse kind of knowledge and does not mean that it is not knowledge *of the world*. Together with his refutation of theocentrism, Allison’s Kant disarms the radical skeptics as well. He does not argue in favor of the belief that we actually *do* have or *might* have ideal knowledge; instead, he uncovers the misleading idea of knowledge that underpins both theocentrism *and* skepticism. Hence, if I’m not wrong in my reading of Allison, from the metaphilosophical perspective it is not the recognition of a non-theocentric notion of knowledge *alone* that makes Kant special, but the recognition in conjunction with *acceptance* of this notion, i.e. acceptance of our epistemic position.

4 Interestingly enough, Putnam, unlike Allison, targets Kant in the same way as he targets Carnap. He claims that Kant is “torn between the idea that all knowledge is partly our own construction” and the idea that knowledge must provide a ‘God-Eye View’. This is due to “the idea that there are *limits to knowledge*” and that we find ourselves in a pickle, facing antinomies “when we try to go beyond those limits” (Putnam 1990: 18). It seems that, for Kant, limits of knowledge are stable, necessary boundaries that cannot be moved.

5 As W. J. Silva-Filho and M. V. Dazzani have aptly pointed out in their recent study, “limits, boundaries, contours, content, are all constructed through the use of language. Borders are events of and in language” (Silva-Filho W. and Dazzani M. 2015: 2).

6 Kołodziejczyk draws on Kant’s idea of spontaneous metaphysics, but for lack of room I do not discuss this in detail; see Kołodziejczyk 2006 [in Polish] and 2009 [in English].

7 I mentioned Plato in the beginning; according to him this stability is guaranteed by the structure of ideas. Thus in the case of a building this could be the mathematical laws taken into account by the architect and engineers (however, Plato’s friend Gorgias would probably have denied the need for any foundations).

8 This is the place where my modest considerations intersect with the huge philosophical stream of thoughts starting from Aristotle, i.e. the stream of thoughts attempting to pick out and express the most fundamental and universal features of reality. I mean the theory (or theories) of categories, providing us with such basic intellectual tools like the distinctions MATTER – FORM, CONCRETE – ABSTRACT, SUBSTANCE – PROPERTY, PART – WHOLE, COMPLEX – SIMPLE etc.; and with such categories as RELATION, CAUSATION, PROCESS, QUALITY, QUANTITY, etc. Let me just mention some of the modern contributors to this field: Charles. S. Peirce (1868), Franz Brentano (1981), Edmund Husserl (1901), Nicolai Hartmann (1938, 1940), Roman Ingarden (1964, 1965a, 1965b); see also Poli (2010), Fine (1992), Perzanowski (1988, 1990).

9 Think of a building again – partition walls can always be, with a bigger or a smaller effort, moved and rearranged, and one can also imagine a smart system in which partitions are moving all the time, in accordance with rules provided by the *inhabitants* of the building. Thus the *inhabitants* of the empirical domain *cast* some of their intellectual tools to play the role of categories. For example, the MENTAL – PHYSICAL pair’s performance is wide of the mark in science according to Searle. By contrast, Ryle seems to accept this duet, but in his view, *within* the MENTAL – PHYSICAL structure, the performance of OBJECT, STATE, PROCESS, etc. is completely misplaced. This, however, does not make them misplaced when it comes to other areas. Hence we can see how much depends on context. Moreover, a priori it is possible that even something like TABLE could be allowed to play the category-role, although this is hardly imaginable in the world as it actually stands for us. It might also turn out that given our cognitive capabilities and the way in which the world appears to us, some items actually must play the category-role, as for example the pair SIMPLE – COMPLEX. However, even if something must play the role, it is still *a role*. Finally, even in the cases of allegedly robust roles there might be surprises: Think for instance of the pair OBJECT – SUBJECT, which has successfully set up the intelligible domain of classical physics but apparently fails to play this role adequately in quantum physics.

10 On the other hand, the Boundary Question of Mind does not *ex definitione* convey any sort of idealism or anti-realism: Minds do *not produce* empirical domains as eyes do not produce their fields of vision.

11 Skepticism outlines an interesting paradox: Our investigation of the frames in which the world is captured is a proper part of the world; paradoxically, investigations of the boundaries of the empirical domain take place inside the empirical domain. Hence, our conceptual investigation of our own epistemic position is constrained by this epistemic position; our investigations of appearances rely on appearances; our investigations of perspective-dependence are perspective-dependent, etc.

12 Skepticism, as it is presented here, is close to the position defended by Colin McGinn (1989), also stressing – in a different manner – the limits of our cognitive capacities.

13 Let me make it clear that I don’t mean *all* kinds of materialism, but only *reductive* materialism.

14 The problem of boundary-*empirical* questions of mind has to do with the controversy over the prospect of naturalizing transcendental and phenomenological analysis (see e.g. Varela 1996; De Preester 2006; Gallagher and Zahavi 2008). There is, however, no place to discuss whether or not naturalization of phenomenology could be done
